# Are non-monetary rewards effective in attracting peer reviewers? A natural experiment

**DOI:** 10.1007/s11192-018-2912-6

**Published:** 2018-09-20

**Authors:** Monica Aniela Zaharie, Marco Seeber

**Affiliations:** 1 Faculty of Economics and Business Administration, Babeş-Bolyai University, 400591 Cluj Napoca, Romania; 2 CHEGG, Department of Sociology, Ghent University, Korte Meer 3, 9000 Ghent, Belgium

**Keywords:** Peer review, Motivations, Non-monetary rewards, Engagement incentives, Task-completion incentives, Performance based incentives

## Abstract

Editors of scientific journals meet increasing challenges to find peer reviewers. Rewarding reviewers has been proposed as a solution to incentives peer review, and journals have already started to offer different kinds of rewards, particularly non-monetary ones. However, research so far has mainly explored the efficacy of monetary rewards, while research on non-monetary rewards is barely absent. The goal of this article is to fill this gap by exploring whether and under what conditions a rather common non-monetary reward employed by journals, i.e., to recognize reviewers work by publishing their names on a yearly issue, is effective in increasing the willingness of scientists to become peer reviewers. We test the efficacy of three different reward settings identified in the literature: (1) engagement contingent, (2) task-completion contingent, and (3) performance contingent, through a natural experiment involving 1865 scientists in faculties of business and economics of Romanian universities. We explore whether reward efficacy varies across scientists depending on their gender, academic rank, research productivity, and type of institution to which they are affiliated. The results show that the performance contingency strongly reduces the number of respondents willing to become reviewers (− 60 % compared to a no-reward setting), particularly males and research productive scientists. Scientists affiliated with private universities are strongly discouraged by the reward. In sum, the results suggest that non-monetary rewards are not necessarily effective, as in some cases they may actually discourage the most intrinsically motivated and competent reviewers.

## Introduction

Peer review is the central practice to select contributions to be published in scientific venues, and one of the processes generating publication traces that are at the heart of bibliometric studies (Squazzoni et al. 2017). Thus far, the scientometrics community has studied peer review as a social process (Squazzoni et al. 2017), through research examining—among others—the role of editors as gatekeepers (Nederhof and Raan 1987), the relationship with bibliometric indicators (Braun and Dióspatonyi 2005), developing models of the peer review process (Ragone et al. 2013; Righi and Takács 2017; Bianchi et al. 2018).

In recent years, research has focused particularly on exploring limitations of peer review and how they can be overcome, in particular examining bias in the review and editorial processes (Lee et al. 2013; Seeber and Bacchelli 2017; Sarigöl et al. 2017). A major concern is also that editors of scientific journals have been facing a growing shortage of peer reviewers, increasing declines to review requests and delays in delivering review reports (Björk and Solomon 2013; Nguyen et al. 2015; Willis 2016). The gap between the number of reviewers needed and those available calls for solutions to attract new referees (Kachewar and Sankaye 2013; Warne 2016). Thus, identifying the impact of certain incentive strategies on scholars’ willingness to become reviewers bears tremendous importance for the quality management of research publications (Azar 2006; Northcraft and Tenbrunsel 2011). In few cases, journals have employed monetary rewards,^1^ while much more common are non-monetary rewards such as awarding a symbolic prize for the best reviewers or publishing the name of the reviewers. Despite the need to understand the effects of incentives on the peer review process, there is limited empirical research (García et al. 2015). Scholarly studies have mostly focused on the impact of *monetary rewards* on the review process (Hamermesh 1994; Chang and Lai 2001; Thompson et al. 2010), some showing potential side effects such as the crowding out intrinsic motivation to peer review (Squazzoni et al. 2013; Zaharie and Osoian 2016). Monetary rewards are problematic for tasks like peer reviews because they harm intrinsic motivation (Ryan and Deci 2000; Gagné and Deci 2005) and voluntarism behaviour (Gneezy et al. 2011). Instead, non-monetary rewards may provide an incentive, while avoiding the drawbacks of monetary rewards (Frey 2007; Gallus and Frey 2016).

Thus far, research on the efficacy of *non*-*monetary rewards* has been limited and barely absent in the context of peer review. Therefore, this article aims to explore whether non-monetary rewards are effective in attracting more reviewers than a baseline setting with no reward. We consider one of the most common forms of non-monetary rewards, i.e., *publishing the name of the reviewer on the journal’s website and issuing a reviewer certificate*, and explore the impact of three reward contingencies on the willingness to review: (1) engagement, (2) task-completion, and (3) performance related. We develop hypotheses on whether the willingness to become a reviewer and the responsiveness to rewards varies along scientists’ gender, academic rank, public-private status of the university to which the scholars are affiliated, and test the hypotheses through a natural experiment involving 1,865 scientists from faculties of business and economics of Romanian universities.

In section “Data and methods” we review the literature on the effects of rewards on peer review and on motivations to peer review, and develop hypotheses on the effect of different reward contingencies on the willingness to become reviewer, as well as on the differential responses of scientists’ according to their gender, affiliation, academic rank and productivity. The third section describes the research design, data, and method. We present the findings in the fourth section and discuss them in the fifth section. In the conclusion section, we reflect on the implications of this study and point out promising directions for future research.

### Theoretical framework

The willingness to perform certain behaviours is related to the characteristics of the action, of the reward setting, and individual idiosyncratic motivations. Hence, in the following paragraphs, we review the debate on the effects of incentives on peer review, the literature on motivations to review and reward contingencies and develop hypotheses on their impact on the willingness to become a reviewer for different subgroups of scientists.

#### Motivations to peer review

Scientists’ most prominent motivations to peer review are enjoyment (intrinsic motivation) and service to the academic community (prosocial motivation), whereas extrinsic motivations^2^—such as increasing the chance of being offered a role in the journal’s editorial team– are much less relevant (Mark Ware Consulting 2008, 2016).

Despite empirical evidence suggesting that extrinsic motivations are weakly important in peer review, rewards are often proposed as an instrument to attract new reviewers (Warne 2016). The kind of rewards that reviewers mention into surveys as more valuable to them are feedbacks from the journal on the usefulness/quality of their review, information on the decision outcome, certificates from the journal and acknowledgment in the journal (Warne 2016). In practice, only few journals pay reviewers, e.g. in relation to the quality and timely of the reviews (van Noorden 2013), while it is more common to offer some sort of non-monetary reward, such as thanking reviewers and recognizing their contribution by publishing their names on the website, offering privileged access to subscription databases and research platforms (Gasparyan et al. 2015), awarding certificates for excellent reviewers (van Dijk 2013), or credits that can be claimed for certain hours of contribution (De Gregory 2004).

It is subject to debate, however, whether reviewers should be rewarded (Engers and Gans 1998), how (Copiello 2018) and whether it would be effective. In this last respect, existing studies have mostly focused on monetary incentives. Hamermesh (1994) found that monetary rewards speed up the peer review process and a timely completion. In a similar vein, Thompson et al. (2010) and Chetty et al. (2014) observed that introducing a reward for completing the report in due time was effective and the length of the report did not decrease. On the other side, Chang and Lai (2001) argue that in order to be effective, payments to reviewers should be inversely proportional to journals’ reputation, so that less reputed outlets would face an unbearable cost. Moreover, Squazzoni et al. (2013) found that offering material incentives to reviewers can have unintended consequences and decrease the quality and efficiency of the reviewing process.

From a scholarly perspective, in classical economic theory rewards are the pivotal tool to encourage a given behaviour, whereas behavioural economists and psychologists have pointed out some potential drawbacks. External rewards can in fact be harmful for behaviours for which prosocial and intrinsic motivations are crucial, by interfering with social norms (Heyman and Ariely 2004; Liberman et al. 2004; Fuster and Meier 2010), undermining trust relationships (Gneezy et al. 2011), reducing the need to signal a voluntarily altruistic act, i.e. “image motivation” (Bénabou and Tirole 2006; Goette et al. 2010), and crowding out intrinsic motivation (Deci et al. 2001).

Alike for peer review research, empirical studies on rewards’ effectiveness have mostly focused on monetary rewards (Heyman and Ariely 2004; Ariely et al. 2009; Fuster and Meier 2010; Bucciol et al. 2015), whereas much less attention has been given to non-monetary rewards. However, some scholars have argued that non-monetary rewards like gifts and awards may provide an incentive for the desired behaviour, while avoiding the drawbacks of monetary rewards (Frey 2007; Gallus and Frey 2016). Therefore, it appears worthwhile to explore the efficacy of similar sort of incentives on the willingness to become a peer reviewer.

#### Impact of the reward setting

Beyond the type of action and reward, how the reward is delivered is arguably very important in affecting the willingness to peer review.

The Cognitive Evaluation Theory (CET) suggests that the impact of external rewards varies as a function of how they influence the individual’s perceived competence and autonomy. For example, verbal rewards may enhance perceived competence, but if they carry a feeling of external control, they will undermine the sense of autonomy and therefore intrinsic motivation (Deci et al. 2001). In terms of extrinsic motivation, *publishing the reviewers’ names* signals to external audiences that a scientist has put an effort in a service to the community, as well as her/his skills-as she/he has been recruited and has performed the task well; these signals can represent valuable reputational payoff for a scientist.

Three categories of reward contingencies have been identified, which have a different impact on perceptions of competence and autonomy (Ryan et al. 1983; Deci et al. 2001), as well as the presence and strength of signals of effort and capability.

*Engagement*-*contingent* rewards are given for the mere agreement to be involved in the task, but there is no requirement to complete the task and therefore little or no controlling effect. Moreover, since the reward is offered for simply engaging in a task, it conveys the irrelevance of personal competence for performing the task. The signals of effort and skills are also very weak because the reviewer obtaining the reward has not necessarily performed the task. In turn, the effects on motivations are likely to be small, and therefore on the willingness to become a reviewer.

*Completion*-*contingent* rewards are given for completing the task. According to CET, such rewards are likely to be perceived as controlling, while they do not enhance the feeling of competence, because the reward is given regardless of the quality or quantity of the review. The reward does communicate that the reviewer has done a service to the community, but it conveys no information about the quantity and quality of such service. Therefore, the completion setting is also expected to have little impact on the adoption of the behaviour.

*Performance*-*contingent* rewards are offered to participants who not only get involved and complete the task, but also achieve high performance. Harackiewicz and Sansone (1991) argue that performance-contingent rewards spur the person to focus on doing the task well (competence valuation). However, this can only occur once the person has received the reward. On the other side, performance-contingent rewards are likely to bear a strong controlling effect, reducing intrinsic motivations. Under a *performance*-*contingent,* the reward carries a much stronger signal, namely that the reviewer has performed a significant effort and in a competent way, and therefore the extrinsic motivations will be increased more than in the engagement and completion contingencies. Given that intrinsic motivations tend to be much more important than extrinsic motivation for peer review, we expect an overall negative effect of the performance contingent setting. We formulate the following hypothesis.

##### Hypothesis 1

Scientists subjected to a performance-contingent reward are less likely to accept to become reviewers than scientists in a no-reward setting.

#### Willingness to review and responsiveness to rewards: differences between scientists

The effectiveness of rewards for different subgroups of scholars has rarely been discussed and explored (Mark Ware Consulting 2008; Zaharie and Osoian 2016). In a recent work, Seeber et al. (2017) found that scientists’ responses to external incentives largely depend on individual strategic considerations. Namely, in response to a new regulation linking career advancement to the number of citations received, scientists increased the number of self-citations as a shortcut to boost their metric scores, particularly if they could benefit from increasing citations, namely scientists with fewer citations and those in need to climb the career ladder. In a similar vein, we expect that the categories of scientists more likely to accept to become reviewers will be those more likely to gain from peer reviewing (e.g. through learning), and the categories of scientists more responsive to the reward—*namely the publication of reviewers’ name and reviewer certificate*—will be those more likely to benefit from their work as reviewers being (publicly) recognized.

In the following paragraphs, we discuss the willingness to become a reviewer and/or the responsiveness to rewards for selected categories of scientists along with their gender, academic rank, research productivity and type of institutional affiliation, as well as develop hypotheses accordingly.

#### Gender

There is no consensus on whether gender is a relevant predictor of the willingness to become a reviewer. For example, an analysis of Publons data found that male reviewers report more reviews compared to female scholars (Ortega 2017), while Lerback and Hanson (2017) found that females are less invited to review, but the gender proportion of reviewers who decline is similar. As a matter of fact, there is no evident reason for why males or females should have different gains from reviewing. From a motivational perspective, males and females have similar intrinsic and extrinsic motivations. On the other side, differences do exist as to the type of prosocial behaviours females and males are inclined to pursue.^3^ However, such differences do not have straightforward implications for predicting the willingness to review.

Regarding variations in the responsiveness to different reward contingencies, research evidence suggests that, when the reward from the competition is large enough, females equal males in their willingness to enter and win the competition (Petrie and Segal 2015), or they are even more responsive. Angrist and Lavy (2009), for example, run an experiment with Israeli High School students where awards were offered to all students who passed their exams, and they observed a substantial increase in certification rates for girls, but no effect on boys.

Such higher responsiveness may be due to the fact that in many contexts women still struggle for emancipation and for being recognized equal status to men. Therefore, an award that recognizes their superior capabilities and effort would have a relatively greater value for them. Hence, we craft the following hypothesis.

##### Hypothesis 2

Under a performance-contingent reward setting, female scientists are more likely to accept to become reviewers than their male peers.

#### Academic rank

The benefits associated with the review are arguably related to the extent to which one is involved in research activity and the extent to which her/his career development depends on research performance. Hence, roles that primarily focus on teaching—in our context of analysis, ‘teaching assistants’ and ‘assistant professors’ (see paragraph 3.3)—are expected to decline more often than academics strongly oriented to research- i.e. associate professors—which would have higher gains both in terms of learning and for career purposes. Full professors are also likely to be weakly motivated, namely because they are expected to be already experienced researchers (Tite and Schroter 2007), they are already at the top of the career ladder, as well as because managerial duties tend to be more important, leaving less time for research and tasks like peer review.^4^ Therefore, we expect that:

##### Hypothesis 3

Academic ranks more focused on research (i.e. associate professors) are more likely to accept becoming reviewers than academic ranks relatively more focused on teaching (i.e. assistant professors and teaching assistant) and managerial tasks (i.e. full professors).

Responsiveness to rewards depends on the relative importance of different type of motivations. For example, if intrinsic motivations are more important than extrinsic motivations, then the reward will have little or even a negative effect. However, it is difficult to argue that intrinsic motivations matter more or less for a given rank. In terms of extrinsic motivations, since peer review has no decisive impact on career advancement, the impact is likely negligible. Hence, no hypothesis can be clearly derived in this regard.

#### Institutional affiliation

In higher education, a notable difference is usually observed between public and private universities, which differ as to their values, norms, and institutional goals (Teixeira et al. 2014). Past research has found that prosocial behaviours are more common among employees in the public sector (Perry and Wise 1990; Dur and Zoutenbier 2014; Bullock et al. 2015). The reason for that may lie in the fact that prosocial activities are rather consistent with the mission of public institutions of serving the society, while they are at odds with the goal of maximizing profit when such prosocial activities do not foresee a tangible return for the organization. However, in normal conditions, peer review is a particular kind of prosocial activity, where, to paraphrase Matthew (6:3) “*the left hand doesn’t know what the right hand does*”, namely it will not be of public dominion. Thus, in the secrecy of the peer review process, there might not be significant differences between scientists of public and private institutions in their willingness to review. On the contrary, disclosing the identity of reviewers by publishing their names is likely to be more welcome in public institutions—signalling the service to the academic community—than in private institutions, as it also signals that the scientist is diverting resources from the organizational goals. Therefore we craft the hypothesis that:

##### Hypothesis 4

Under a reward setting that consists in publishing the name of the reviewers, scientists affiliated with private universities are less likely to accept to become reviewers than their peers affiliated to public universities.

#### Scientific productivity

The relationship between scientific productivity and willingness to review is uncertain. On the one hand, highly productive scientists are likely to be those enjoying the most doing research (strong intrinsic motivations) and hence likely to enjoy reviewing as well; on the other hand, highly productive scientists will be also more skilled and therefore gain less in terms of learning through peer review. Therefore, no straightforward expectations emerge regarding the willingness to review in a no-reward setting. On the contrary, because of the stronger intrinsic motivations that arguably characterize high productive scientists, we expect them to be less responsive to rewards, especially in the case of performance-related ones, as they might enhance pressure and diminish the enjoyment of the task.

##### Hypothesis 5

Under a performance-contingent reward setting, scientists with higher productivity will be less likely to accept to become reviewers.

## Data and methods

### Experiment

This study is based on a natural experiment run by *Studia Universitatis Babeş*-*Bolyai Oeconomica*, a scientific journal in Business and Economics, aiming to expand its pool of reviewers. Starting from the findings of peer review surveys on the most preferred incentives (Mark Ware Consulting 2008; Warne 2016), the editorial team decided to encourage scientists to become reviewers for the journal by using a rather common reward offered by journals: acknowledgment in the journal and the issuance of a Review Certificate upon request. These rewards recognize the review process and are kind of ribbon category of scientists’ motivations (Lam 2011).

A pool of scientists was contacted by email and asked if they were willing to become part of the pool of reviewers for the journal (see the text of the emails in the appendix). In case of acceptance, and to measure the extent to which they were willing to put an effort in peer review for the journal, they were also asked how many articles they were available to review each year and the amount of time—i.e. how many months - they foreseen to provide a review report.

Four experimental settings—groups were tested:

- *No*-*reward*: no reward was offered for accepting the invitation to become reviewers
- *Engagement*: reward for accepting to become part of the pool of reviewers
- *Completion*: reward for completing at least a review report
- *Performance*: reward for quality and quantity of reviews

### Journal and sample

The journal *Studia Universitatis Babeş*-*Bolyai Oeconomica* is the official publication of the Faculty of Economics and Business Administration, Babeş-Bolyai University, which is one of the largest, oldest and highest ranked universities in Romania. The journal is included in the official list of journals, indexed in international databases (EconLit, Ebsco, Proquest, CEEOL, and IBSS), hence having a medium-to-high status in this context. The journal is published in English and adopts a double-blind peer review model, with three issues per year, five papers per issue, with an average rejection rate of 40%. The choice of the journal was driven by conceptual and empirical considerations. A key condition for the experiment is that the non-monetary reward should be sufficiently important to make a difference in the response rate. The selected journal holds a medium-high prestige in the Romanian economists’ community, which makes it an appropriate case. Moreover, the journal was aiming to expand its pool of reviewers and find the most effective ways in doing that, and one of the authors is a member of the editorial board, hence providing the opportunity to run the experiment under optimal conditions of control.

The pool of potential referees contacted for the experiment included scientists in business and economics from Romanian universities. The email addresses and the academic ranks were extracted from the official web page of the institution. From the total 99 Romanian universities across all fields, scientists from 35 universities—40 faculties—offering programs in economics and business were included in the study. In total, there were collected the contact details for 1946 scholars. Names were eliminated when double listed due to the affiliation to more institutions (9) or due to inconsistencies related to the way the names were listed on the websites (14). Since the previous experience with the journal could influence the decision to review, the scientists who had published or submitted a manuscript in the past 5 years, as well as scientists that had already been part of the reviewers’ pool (58) were not included in the study. The final pool of potential reviewers included 1865 scientists, of which: 87.2% from public universities, 57.3% females, and 25.4% full professors, 33.1% associate professors, 30.5% assistant professors, and 10.8% teaching assistants. We assigned individuals to the four experimental settings in order to obtain similar proportions of respondents by faculty, type of university, academic rank, and gender.

### Context of analysis

Romania is part of the European Union since 2007. The higher education system includes 99 universities (56 public and 43 private universities) that employ over 26,949 scientists and enrols 535,200 bachelor and master students (Ministry of Education 2015, 2016). Remarkable differences exist between public and private universities. Public universities are financed mostly from the national budget, while private universities do not receive any public funds. Moreover, compared to public universities, private universities tend to be more oriented to teaching than research (Ministry of Education, Research, Youth, and Sports 2011a, b). Emphasis on research has grown throughout time, particularly in public universities. In 2005 the government introduced a set of minimum standards based on scientific publications for attaining higher academic ranks, and these standards have frequently been updated. As such, peer review is not considered as a criterion for promotion, yet since 2011, research evaluation criteria also include the membership in journals’ editorial boards.

### Variables

#### Dependent variables

The first dependent variable considers whether the contacted person opened the email or not. We regard this variable as a proxy for the general interest in becoming a reviewer. In fact, the “object” of the mail was “Invitation to become a reviewer for *name of the journal*”, so that the purpose of the message was rather evident.

The second dependent variable considers whether the contacted person accepted to become a reviewer (1) or not (0). The sample includes scholars who opened the email and accepted within the first day after receiving the email.^5^ We chose to consider those opening and accepting on the same day of receiving the invitation in order to minimize the risk that scientists became aware of other experimental settings, which would have reduced the validity of the experiment.^6^ We used the MailChimp email service, which allows the identification of the recipients who did not open the email invitation.

#### Independent variables

##### Reward setting

A categorical variable considers whether the potential reviewer was contacted employing a no-reward, engagement, completion or performance setting.

##### Type of institutional affiliation

A dummy variable takes into consideration the affiliation with a public or private university.

##### Gender

A dummy variable for the gender of the respondent. The number of female scientists in Romanian universities increased in recent decades, and currently represent 49.4% of the academic body, although only 30.2% of the full professors.

##### Academic rank

A categorical variable considers the scientist’s academic rank, namely: teaching assistant, assistant professor, associate professor, and full professor. In the Romanian context, the duties of teaching assistant and assistant professors mostly focus on teaching, associate professors are on average more oriented to research, whereas full professors spend much time on managerial tasks.

##### Research productivity

For scientists opening the email, we collected three scores of scientific productivity from ISI Web of Science: (1) the number of articles, (2) the number of proceeding articles and (3) the Hirsch index of citations (Hirsch 2005). The three productivity scores are significantly correlated with one another. A factor analysis extracts one factor absorbing 73% of the variance, which we employ as a variable of scientific productivity, rescaled to range between 1 and 11.4.

#### Methods

The empirical analysis combines descriptive and inferential statistics.

First, we describe the scientists’ propensity to accept to review along the main predicting variables, as well as the ‘promised’ effort in terms of time to review and the number of manuscripts.

Next, we run regression models to test the factors predicting the chance of opening the email and the willingness to become reviewer. Since the dependent variables are binary—i.e., open (1) or not open (0) the email, accept (1) or not accept (1) to become a reviewer—we employ logistic regressions.

#### Assumptions of logistic regressions

Logistic regressions require four major conditions to be satisfied (Garson 2016).

First, the observations to be independent of each other. Therefore, we considered for the analysis only those scientists responding the same day they received the email, in order to avoid that scientists replied only after learning of the invitation from other colleagues.

Second, multicollinearity is an issue in (logistic) regressions when two or more predicting variables are strongly associated. A cross tabulation of the frequency of the main predicting variables highlights that scientists are very similarly distributed along the different cross-categories, which suggest that multicollinearity should not be an issue (see Table 5 in the appendix). Small differences are also observed in the research productivity by gender (females: mean 1.75, median 1.51; males: 1.89 and 1.53), and university type (public universities: mean 1.87, median 1.57; private universities: mean 1.38, median 1.19), although some notable differences are observed in the research productivity e.g. between full professors and teaching assistant (Table 6 in the appendix). Therefore, we computed the Variance Inflation Factors (VIF) as a standard test for multicollinearity (values should be below 10). The VIF values are all very low (below 1.15), which excludes multicollinearity among the predicting variables.

Third, logistic regression typically requires large samples. A common rule of thumb is a minimum of ten cases with the least frequent outcome for each independent variable in your model. This study explores five independent variables and the expected probability of the least frequent outcome is 0.3 which means (10*5/0.30) = 300, whereas the samples include 1,865 and 735 units.

Finally, logistic regression assumes linearity of independent variables and log odds- specifically for independent continuous variables. Most of the independent variables are categorical. We run the Box-Tidwell test by including in the model the interaction between the research productivity and its logs, and it is not significant, meaning that the assumption is not violated (Garson 2016).

In sum, all the four conditions are satisfied.

#### Model estimation and fit measures

The sample for the second analysis of acceptance is not randomly selected as it is represented by scientists that in the first place opened the email. A Heckman correction would then be appropriate (Heckman 1979): a two-step statistical approach that corrects for non-randomly selected samples. However, the model does not converge. In any case, the only significant difference in opening emails is related to rank—as associate professors are more likely to respond. Hence our choice to run two separates models.

We run multilevel models to take into considerations that scientists (level 1) are nested into different faculties (level 2), as neglecting a multilevel structure may lead to biased coefficients (Robinson 2009). Since for this type of models maximum-likelihood estimates provide biased results, we estimate the model through Bayesian Markov Chain Monte Carlo methods (MCMC) (Snijders and Bosker 2012). Bayesian statistics’ interpretation of probability expresses a degree of belief in an event, based on e.g. prior knowledge about the event, and differs from the frequentist probability, which sees probability as the limit of the relative frequency of an event after a large number of trials. Bayesian statistics is computationally burdensome and has become popular with the growth of computational power and computing algorithms, such as MCMC, which is a simulation-based algorithm producing a sequence of values that converge to the posterior distribution. In our models, the starting estimates derived from a maximum likelihood procedure, to which a MCMC Bayesian estimation followed.

As a diagnostic for model comparison we employ the Deviance Information Criterion (DIC), which penalizes for a model complexity—similarly to the Akaike Information Criterion (AIC) and it is a measure particularly valuable for testing improved goodness of fit in logit models (Jones and Subramanian 2012). The Akaike Information Criterion—AIC (Akaike 1974) compares models by considering both goodness of fit and complexity of the model, estimating the loss of information due to using a given model to represent the true model, i.e. a hypothetical model that would perfectly describe the data. Accordingly, the model with the smaller AIC points out the model that implies the smaller loss of information, thus having more chances to be the best model. In particular, given n models from 1 to n models and model_min_ being the one with the smaller AIC, then the exponential of (AIC_min_AIC_j_)/2 indicates the probability of model_j_ in respect to model_min_ to minimize the loss of information.

In order to assess the goodness of fit of the regression model, we computed the predicted probability of accepting to review from the regression model with the main interactions. Next, we considered different thresholds of probability for determining whether the scientist accept (positive values = 1) or not (negative values = 0) to review. Third, we computed for each probability threshold two standard measures of fit for logistic regressions: sensitivity and specificity. *Sensibility* measures what share of actual positives that are correctly identified as such, and it is given by the ration between values predicted as positive which are true positive divided by the total number of positive (accepting to become reviewer). *Specificity* measures the share of actual negatives that are correctly identified as such, and it is given by the ration between values predicted as negative which are truly negative divided by the total number of negative (refusing to become reviewer).

### Data analysis

#### Descriptive statistics

The sample includes 1865 scholars who received the email invitation to become a reviewer. 735 scientists (39.4%) opened the email the same day, and among them, 306 (41.6%) accepted to become reviewer the same day.

Table 1 displays the proportions of contacted scholars who opened and accepted to review along the selected independent variables. In terms of opening the email, the associate professors opened the email more frequently than the other academic ranks, while differences by gender and institutional affiliation are very small. Among those who opened the email: female, associate professors, and scientists employed in public universities are more likely to agree to become reviewers. The experimental setting was only visible after opening the email. Therefore, the small variations between experimental groups in opening the email are due to chance. Much more remarkable are the differences in the acceptance rates among those opening the email. These show a higher acceptance rate for the engagement and completion settings and a considerably lower acceptance rate for the performance reward setting.^7^

**Table 1:** Proportions of scientists opening the email and accepting to become reviewers along the main predictors

	Opening (1st day) (%)	Of those opening: accept (1st day) (%)
*Experimental group*		
Baseline	38.0	41.8
Accepting	42.8	44.5
Accomplishing	38.6	47.0
Performance	38.2	32.8
*Gender*		
Female	38.8	44.7
Male	40.3	37.7
*Academic rank*		
Full professor	38.1	35.6
Associate professor	47.2	47.8
Assistant professor	34.7	41.4
Teaching assistant	32.2	31.8
*University type*		
Public	39.4	42.4
Private	39.2	36.6

A closer examination of the acceptance rates by combining experimental settings and control variables provides additional insights (Table 2). Females are more responsive than males under the reward settings, particularly under the performance contingency. The performance setting is generally less attractive for all the academic ranks, with partly the exception of teaching assistants (although the number of cases is small). Scientists from private universities are much more likely to accept in the no-reward setting than in the reward settings, whereas the contrary is true for scientists working in public universities (except for the performance setting). Scientists who accept to become reviewers are not significantly more productive than scientists who do not accept, regardless of the experimental setting (one way ANOVA tests).

**Table 2:** Acceptance rate by experimental setting and main variables

	Gender (%)		Academic rank (%)				University type (%)		Research productivity (%)
	Female	Male	Full professor	Associate professor	Assistant professor	Teaching assistant	Public	Private	Ratio: median prod. accepting yes/no
Baseline	40	43	36	49	46	13	39	59	1.04
Accepting	47	41	32	50	55	31	45	39	1.00
Accomplishing	50	43	44	54	43	41	50	25	0.97
Performance	40	23	32	38	25	38	34	25	1.08

Table 3 suggests that when scientists foresee more effort (completion and performance settings) then they also foresee slightly more time for returning a review report. In terms of the number of manuscripts, scientists are willing to review a smaller number in the performance setting—arguably because more effort is required to produce a high-quality report—whereas the number of manuscripts is the highest for the engagement reward setting. However, the differences between the four settings are not statistically significant (Kruskal–Wallis non-parametric test *p* value 0.105 for time for review and *p* value 0.471 for the number of manuscripts), suggesting that the impact of the reward is negligible in these regards.

**Table 3:** Effort in peer review

	*n* manuscripts		Time	
	Mean	SD	Mean	SD
Baseline	5.07	3.25	1.37	0.67
Accepting	5.30	3.63	1.37	0.54
Accomplishing	4.96	3.99	1.52	0.55
Performance	4.62	3.73	1.49	0.57

### Regression analysis

Table 4 presents the results of five multilevel binary regressions.

**Table 4:** Regression analysis: open email and accept to become reviewer

Fixed part	Open email			Accept: experimental			Accept: main variable			Accept: main interactions			Accept: exp * productivity		
	Coeff.	S.E.	Sign.	Coeff.	S.E.	Sign.	Coeff.	S.E.	Sign.	Coeff.	S.E.	Sign.	Coeff.	S.E.	Sign.
Cons	− 0.674	0.16	***	− 0.27	0.2	**–**	− 0.53	0.28	–	− 0.46	0.32	**–**	− 0.49	0.32	
Exp: engagement versus baseline				0.1	0.22	**–**	0.07	0.22	**–**	0.01	0.34	**–**			
Exp: completion versus baseline				0.25	0.21	**–**	0.25	0.23	**–**	0.19	0.35	**–**			
Exp: performance versus baseline				− 0.44	0.23	**–**	− 0.44	0.24	–	− 0.93	0.37	*			
Gender: female versus male	− 0.011	0.1					0.34	0.16	*	− 0.21	0.33	**–**	0.34	0.16	*
Rank: associate versus full professors	0.498	0.13	***				0.41	0.21	–	0.47	0.22	*	0.40	0.21	**–**
Rank: assistant versus full professors	− 0.032	0.14	**–**				0.11	0.23	–	0.15	0.23	**–**	0.10	0.24	**–**
Rank: teaching assistant versus full *p*.	− 0.147	0.19	**–**				− 0.33	0.33	–	− 0.3	0.33	**–**	− 0.38	0.33	**–**
Uni type: private versus public	− 0.054	0.24	**–**				− 0.51	0.35	–	0.81	0.57	**–**	− 0.54	0.40	
Engagement * female versus male										0.45	0.46	**–**			
Completion * female versus male										0.65	0.47	**–**			
Performance * female versus male										1.23	0.49	*			
Engagement * private versus public										− 1.34	0.7	**–**			
Completion * private versus public										− 2.3	0.73	**			
Performance * private versus public										− 1.65	0.75	*			
Baseline * Research productivity													− 0.01	0.10	**–**
Engagement * research productivity													− 0.01	0.11	**–**
Completion * research productivity													0.09	0.13	**–**
Performance * research productivity													− 0.21	0.12	**–**
*Random part*															
Level: id_uni	0.251	0.11	*	0.33	0.18	**–**	0.36	0.19	**–**	0.41	0.21	**–**	0.37	0.21	**–**
DIC:	2430.2			968.7			963.3			958.6			968.5		
pD:	27.3			20.3			25.0			32.0			26.5		
Units: id_uni	40			36			36			36			36		
Units: id_case	1865			735			735			735			735		

Signif. codes: 0 ‘***’ 0.001 ‘**’ 0.01 ‘*’ 0.05 ‘.’ 0.1 ‘’ 1

In the first model, the dependent variable is whether the email was opened or not. The main control variables are included. The results show that associate professors are significantly more likely to open the email, which indicates a higher interest in becoming reviewers. Gender and institutional affiliation are not significant predictors.

Four models explore the predictors of accepting to become a reviewer.

The second model only includes the experimental setting variable and shows that under the performance-reward setting scientists are less likely to accept (marginally significant, *p* value 0.056).

The third model includes the main predicting variables and shows an overall higher acceptance rate of female scientists.

The fourth model also explores the interactions between the experimental settings and the predicting variables. There are no significant differences between the no-reward setting and the engagement and completion reward settings. On the other hand, respondents are significantly (*p* value 0.012) and strongly less likely (− 60.4%)^8^ to accept becoming a reviewer in a performance setting than in a no-reward setting, which corroborates Hypothesis 1. There are no significant differences in acceptance between females and males in the no-reward, engagement and completion settings, while under the performance rewards setting women are strongly more likely than men to accept becoming reviewers (3.42 times more likely) (Hypothesis 2 supported). Associate professors accept significantly more frequently than full professors, whereas there are no differences between full professors, assistant professors and teaching assistant, which is consistent with Hypothesis 3. There are no significant differences between scientists from public and private universities in the no-reward setting, while coherently with Hypothesis 3, scientists from private universities are less likely to accept in the reward settings than scientists from public universities (up to − 90%). DIC values highlight the better fit of the fourth model with respect to models 2, 3 and 5.

The fifth model explores the interactions between experimental settings and research productivity, showing no significant effects in the no-reward, engagement and completion settings, and a marginally significant and negative effect in the performance setting (*p* value 0.08) (Hypothesis 4 partly supported).

**Fig. 1:**
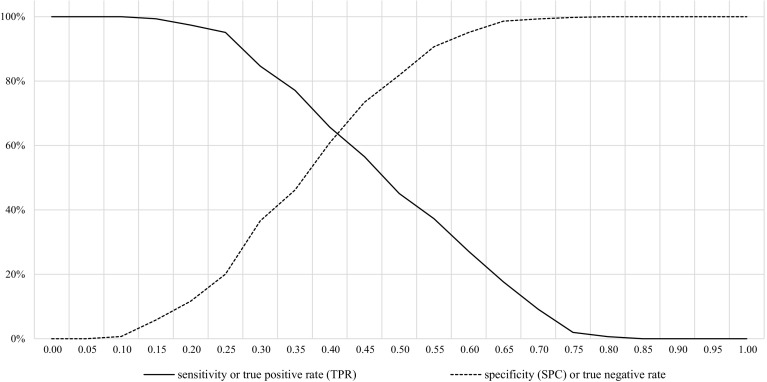
Sensitivity and specificity measures of statistical performance for varying probability thresholds

Figure 1 illustrates sensitivity and specificity scores for different probability thresholds. The predicting power of the model is particularly good around a probability threshold of 0.45: sensitivity (57%), specificity (73%).

## Discussion

This paper explores the efficacy of a non-monetary reward–i.e., acknowledgment of the reviewers’ name in the journal and issuance of a reviewer certificate—and three reward settings in attracting peer reviewers. While studies on motivations have extensively discussed the negative effects of monetary rewards for intrinsically motivated activities (Heyman and Ariely 2004; Ariely et al. 2009; Fuster and Meier 2010), it is often taken for granted that non-monetary incentives have positive effects. These expectations have been backed so far also in the context of peer review, particularly by survey findings showing that reviewers appreciate non-monetary rewards, such as receiving feedback on the usefulness and quality of their review, certificates from the journal and acknowledgment in the journal (Sense about science 2009; Warne 2016). However, the results of the analysis show that acknowledging the work of the reviewers—a rather common non-monetary reward—is not effective in attracting more reviewers, and that the performance setting has a strong negative impact on the acceptance rate (− 60%). We argued that performance-based rewards are likely perceived to have a controlling effect and to exert pressure on the reviewer, which is counterproductive for participation in a voluntary activity mostly driven by enjoyment and prosocial motivations.

The impact of the reward varies considerably across categories of scientists. Female scholars are more responsive than males in a performance setting, arguably because a reward that signals superior capability and effort is particularly attractive for categories of people who have traditionally been a minority and hold lower status positions. Scientists affiliated to private universities are strongly less responsive than public universities scientists in all the reward settings because publishing their names would reveal that they are spending their time in prosocial activities, which are at odds with the key goal of private organizations to maximize profit. Under a performance setting, highly productive scientists also appear to be less responsive.

These findings suggest that rewards—particularly in a performance contingency—may unleash an adverse selection process, by attracting extrinsically motivated reviewers and discouraging scientifically productive, intrinsic and prosocial motivated ones. Given that intrinsic motivation increases curiosity and cognitive flexibility, which are pivotal for complex tasks that require deep information processing (e.g. Gagné and Deci 2005), then such subtle selection effect can have negative consequences on the quality of peer review.

The results bear value by providing editors with empirical evidence on the efficacy of non-monetary rewards, and the interactions with scientists’ characteristics. Similar rewards have been adopted without their efficacy in boosting peer review and possible unintended effects being carefully assessed.

## Conclusions

This article aimed to fill a gap in our understanding of whether rewards can be beneficial for peer review, and particularly non-monetary rewards. We explored whether and under what conditions recognizing reviewers’ work by publishing their names increases the willingness of scientists to become peer reviewers. We tested the efficacy of three different reward settings through a natural experiment involving 1865 scientists in faculties of business and economics of Romanian universities. The results show that this reward is not effective and that under the performance contingency the number of respondents willing to become reviewers even decrease strongly, particularly for research productive scientists. In turn, the effects of non-monetary rewards should not be assumed to be positive, as they may end up discouraging precisely the most intrinsically motivated and competent reviewers.

This study was limited to one form of non-monetary reward, the specific setting of Business and Economics researchers in Romania, and on the effects on the willingness to become a reviewer. Future studies should explore the efficacy of other forms of non-monetary rewards, such as giving feedbacks to reviewers, keeping them updated on the outcome of the review, becoming part of the editorial team, as well as the effects of rewards on the number, timely and quality of review reports. Future research should also bear in mind that peer review surveys identified differences between disciplinary fields with regard to the declared preferred rewards (Sense About Science 2009; Mark Ware Consulting 2008, 2016), that differences between private and public institutions may be smaller—or greater—in other countries, that journals’ status may affect the responsiveness to different reward contingencies.

## Appendix

**Table:** Body of the invitation sent to the scientists as a potential pool of reviewers (Tables 5, 6)

Dear [academic rank and name],
The editorial board of the business and economics journal”…” is currently expanding its team of reviewers. The journal is an official publication of the…. It is an open access journal that does not charge authors nor readers, and it is indexed in international databases EconLit, Ebsco, Proquest, CEEOL, and IBSS.
Given your expertise in the field, we would appreciate if you agree to become part of the pool of referees so that we can contact you in the future to review manuscripts.
[Setting 1: To recognize the reviewers, the scholars who become part of the team of reviewers will be issued a Reviewer Certificate and their names will be published yearly on the journal website.]
[Setting 2: To recognize the reviewers, the scholars who review at least one manuscript during the previous year will be issued a Reviewer Certificate and their names will be published on the journal website.]
[Setting 3: To recognize the review performance, the journal will award the Certificate of Excellence in Reviewing to the scholars who contributed the most to the review process during the previous year, in terms of quantity and quality of the reviews, as assessed by the editorial board. Also, the names of the Excellence Reviewers will be published on the journal website.]
If you ACCEPT to become a reviewer for our journal, click this confirmation **LINK**.
If you DO NOT ACCEPT to become a reviewer for our journal, click this **LINK**.
For any questions, please do not hesitate to contact us at…
With kind regards,
The Editorial Team of the journal

**Table 5:** Scientists by gender, academic rank and university type

	Academic rank				Gender	
	Assistant professor (%)	Associate professor (%)	Full professor (%)	Teaching assistant (%)	Female (%)	Male (%)
*University status*						
Private	33	35	19	12	58	42
Public	30	33	26	11	57	43
*Gender*						
Female	34	33	19	13		
Male	25	33	34	8

**Table 6:** Research productivity of scientists by university type, academic rank and gender

	Research productivity	
	Mean	Median
*University type*		
Private	1.38	1.19
Public	1.87	1.57
*Academic rank*		
Assistant professor	1.5	1.26
Associate professor	1.79	1.53
Full professor	2.32	2.05
Teaching assistant	1.41	1.15
*Gender*		
Female	1.75	1.51
Male	1.89	1.53

## Footnotes

1 E.g. the journals published by the American Economic Association, Colabra: Psychology.

2 People doing an activity are intrinsically motivated when they find the activity interesting and derive spontaneous satisfaction from the activity itself, whereas they are extrinsically motivated when they perceive the activity an instrument to achieve tangible or verbal rewards (Gagné and Deci 2005).

3 Women are more inclined to involve in actions that are rather communal and focused on close, dyadic relations, while males in more agentic, collective-focused, and strength-intensive actions (Eagly 2009).

4 Stossel (1985) also found that most review declinations come from reviewers with a high academic rank and/or reputation.

5 The invitation was sent once, without reminders.

6 Given a one-day limit some scientists did not have sufficient time to reply, e.g. because out of office for a conference. Nevertheless, the goal of the article is not to ascertain what is the acceptance rate, but differences in acceptance rate under different incentive settings. Variations in the non-availability to respond within a day may correlate so some traits, such as the academic rank; in any case, we ensured that experimental groups members have similar traits so it is very unlikely that non-availability rate varies between the experimental settings. In fact differences between experimental settings are similar when the overall acceptance rate is considered (see footnote 7).

7 We controlled whether the lower acceptance rate for the performance reward setting was only expressive of an attitude of the respondents or also due to the one-day limit, since peoples asked to review in a performance setting may take more time to respond. We compared the share of those accepting to review the same day and all those accepting. The number of those accepting increases the most for the baseline (from 42 to 55%) and performance experimental setting (33–46%), and slightly less for the accepting (45–56%) and accomplishing (47–56%). Therefore, the performance setting is in both cases substantially less effective in attracting new reviewers than the other settings.

8 Estimated odds for logistic regressions are computed with following formula: exponential (coefficient), hence e^(−0.933)^ = 0.396.
